# Impact of oil-based contrast agents in hysterosalpingography on fertility outcomes in endometriosis: a retrospective cohort study

**DOI:** 10.1186/s12958-024-01190-1

**Published:** 2024-02-03

**Authors:** Baoli Xie, Yingqin Huang, Fu Hang, Jiaxin Yu, Qianwen Hu, Jiaxu Li, Aiping Qin

**Affiliations:** 1 Center of Reproductive Medicine, The First Affiliated Hospital of Guangxi Medical University, Nanning, 530021 China; 2 Center for Reproductive Medicine, Maternal and Child Health Hospital in Guangxi, Guangxi, 530021 China

**Keywords:** HSG, Oil-based contrast medium, Fertility enhancement, Infertility, Endometriosis, Propensity score matching(PSM)

## Abstract

**Background:**

Previous studies have suggested that oil-based contrast agents used during hysterosalpingography (HSG) in infertile patients can enhance fertility. However, limited research has investigated the effect of oil-based contrast medium specifically in individuals with endometriosis-related infertility.

**Objective:**

This study aims to explore the impact of oil-based contrast medium on fertility outcomes in women with endometriosis-related infertility.

**Methods:**

Conducted at the First Affiliated Hospital of Guangxi Medical University (January 2020 to June 2022), the study included 512 patients undergoing HSG. Patients were categorized into oil-based and non-oil-based groups, and after propensity score matching, demographic characteristics were compared. Main outcomes included clinical pregnancy rates, live birth rates, early miscarriage rates, and ectopic pregnancy rates.

**Results:**

In our analysis, the Oil-based group showed significantly better outcomes compared to the Non-oil-based group. Specifically, the Oil-based group had higher clinical pregnancy rates (51.39% vs. 27.36%) and increased live birth rates (31.48% vs. 19.93%). This trend held true for expectant treatment, IUI, and IVF/ICSI, except for surgical treatment where no significant difference was observed. After adjusting for various factors using propensity score matching, the Non-oil-based group consistently exhibited lower clinical pregnancy rates compared to the Oil-based group. The Odds Ratio (OR) was 0.38 (95%CI: 0.27–0.55) without adjustment, 0.34 (0.22–0.51) in multivariable analysis, 0.39 (0.27–0.57) using inverse probability of treatment weighting (IPTW), and 0.22 (0.14–0.35) in propensity score matching.

**Conclusion:**

Oil-based contrast medium used in HSG for women with endometriosis-related infertility is associated with higher clinical pregnancy rates and live birth rates compared to Non-oil-based contrast medium.

## Background

Approximately one in six couples seeking to conceive experiences infertility, defined as the inability to conceive after a year of unprotected sexual activity [1]. Hysterosalpingography (HSG) has emerged as a promising approach to enhance female fertility. Initially used for the screening and diagnosis of infertility [2], the expanding use of HSG among infertility patients has highlighted its potential to improve fertility outcomes [3–5]. However, the efficacy of this fertility-enhancing impact varies depending on the contrast agent used during the HSG procedure [6]. Some researchers [7]have suggested that oil-based contrast agents may exhibit superiority over water-based contrast agents in enhancing fertility among infertility patients, possibly through mechanisms such as modulating T-cell immunity, altering membrane polarity, viscosity, and washout effects.

Endometriosis is a challenging condition estimated to affect one in every ten reproductive-age women globally [8]. As a prevalent gynecological condition, the prevalence of endometriosis surged dramatically to 10% in 2020, and approximately 50% of women with endometriosis experience recurrent symptoms over a 5-year period, irrespective of the treatment approach [9, 10]. Inflammation and fibrosis, induced by endometriosis, lead to the development of endometrial tissue outside the uterus, causing recurrent bleeding. This results in symptoms such as chronic fatigue, dysmenorrhea, dyspareunia, bladder and bowel endometriosis, and chronic pelvic discomfort [11]. Endometriosis is estimated to contribute to 30-50% of incurable infertility cases, posing a significant concern among reproductive-age patients [12]. Infertility rates have been steadily rising over the past few decades, ranging from 13.00 to 24.58% in women desiring fertility [13, 14]. Typically, due to delayed diagnosis, this form of infertility remains unexplained, subjecting patients to significant social, psychological, and reproductive pressures [15]. Thus, there is an urgent need for a novel treatment strategy to address infertility caused by endometriosis.

A growing body of evidence suggests that the use of iodinated oil contrast agents can improve the fertility of women experiencing infertility [3, 16, 17]. The consensus statement published by the Australian Reproductive Endocrinology and Infertility Consensus Expert Group in 2020 [18] emphasized that, compared to water-soluble contrast agents, women with unexplained infertility undergoing HSG using oil-based contrast agents have a higher likelihood of achieving and sustaining pregnancies, receiving a Grade 2 recommendation. However, some studies have also explored whether the administration of iodinated oil, particularly Iodine Oil®, prior to in vitro fertilization (IVF), enhances the success rates of IVF treatments for women with conditions like endometriosis or recurrent implantation failure (RIF) [19]. These trials had limited statistical power to detect small differences between the treatment and control groups.

In a significant randomized controlled trial (RCT) conducted in the Netherlands in 2017, it was found that six months post-operation, oil-based contrast agents increased the pregnancy rate by 10% compared to water-soluble contrast agents among the general infertility population [3]. However, this study excluded patients with conditions like endometriosis and polycystic ovarian syndrome, among others. While there is a consensus regarding the effectiveness of iodinated oil in improving fertility among the general infertility population, it remains uncertain whether iodinated oil HSG offers similar advantages to patients experiencing infertility associated with endometriosis.

To address this question, we conducted a retrospective study comparing the impact of iodinated oil and iodinated water contrast agents on pregnancy outcomes in patients with infertility linked to endometriosis.

## Methods

### Study design

This study included individuals who underwent HSG due to infertility combined with endometriosis at the First Affiliated Hospital of Guangxi Medical University between January 2020 and June 2022. The research adhered to the guidelines outlined in the STROBE Statement. Approval for the study protocol was obtained from the Ethics Committee of the First Affiliated Hospital of Guangxi Medical University. The inclusion criteria were as follows: (1) Female patients with endometriosis aged between 18 and 39 years old; (2) Having a natural menstrual cycle; (3) Having undergone HSG. The exclusion criteria were: (1) Female participants with uncontrolled endocrine disorders known to diminish natural pregnancy chances (e.g., the acute phase of systemic lupus erythematosus); (2) Total motile sperm count after sperm wash of less than 3 million sperm per milliliter in the male partner (or a total motile sperm count of < 1 million sperm per milliliter when an analysis after sperm wash was not performed); (3) Participants with incomplete data. The study was approved by the Ethics Committee of First Affiliated Hospital of Guangxi Medical University. Patients’ basic parameters included age, complication, BMI, duration of infertility, smoking status, surgical history, total number of previous, pregnancies resulting in live births, miscarriage times, treatment after HSG and duration between HSG and pregnancy.

### Definition of endometriosis

Endometriosis was defined based on at least one of the following conditions: (1) confirmation by surgery: endometriosis was confirmed through laparoscopic or transabdominal surgery; (2) identification by ultrasonography: an endometrioma was discovered using transvaginal ultrasonography. A typical endometrioma manifests as a single or multilocular (fewer than five locules) cyst with ground glass echogenicity of the cyst fluid [20]; (3) clinical Indicators of suspected endometriosis: suspected endometriosis could be established based on the presence of any three of the following five factors: infertility, dysmenorrhea, dyspareunia, sacral ligament discomfort, CA125 levels exceeding 15 mIU/mL [21].

### Main outcomes

The primary endpoint for clinical pregnancy was determined as the first day of the final menstrual cycle within a year of HSG. Clinical pregnancy was defined as the presence of a gestational sac detected through ultrasonography. Secondary outcome measures included: (1) live Birth: defined as a live birth occurring after 28 weeks of pregnancy; (2) miscarriage: defined as the absence of a fetal heartbeat on ultrasound or a spontaneous loss of pregnancy occurring before 12 weeks of pregnancy; (3) ectopic Pregnancy: defined as an embryo implanted outside the uterus.

### Statistical analysis

Data analysis was conducted utilizing SPSS software, version 27.0 (IBM), and R software, version 3.3.1 (R Project for Statistical Computing). For categorical data, rates were expressed, while quantitative data were presented as the mean standard deviation (SD). Chi-square analysis was employed to assess differences among categorized data groups. To compare the cumulative pregnancy rates across different groups, the Kaplan-Meier curve was utilized. Statistical significance was considered when *p* < 0.05 was reached. Given the non-randomized study design, a matched propensity score (PSM) analysis was performed to assess covariates. The PSM was estimated using multivariable logistic regression. Age, complications, BMI, duration of infertility, smoking status, surgical history, total number of previous pregnancies resulting in live births, miscarriage times, and treatment after HSG were considered potential confounders. Other parameters used in the study were also included as independent variables for PSM in the present analysis. The logit-transformed PS matching was performed using a 1:1 ratio protocol without replacement (greedy-matching algorithm) with a caliper width of 0.2 standard deviation. Balance of covariates was judged by standardized differences. Here the balance is considered to be satisfactory when the standardized difference is less than 10% [22].

## Results

### Clinical features

**Fig. 1:**
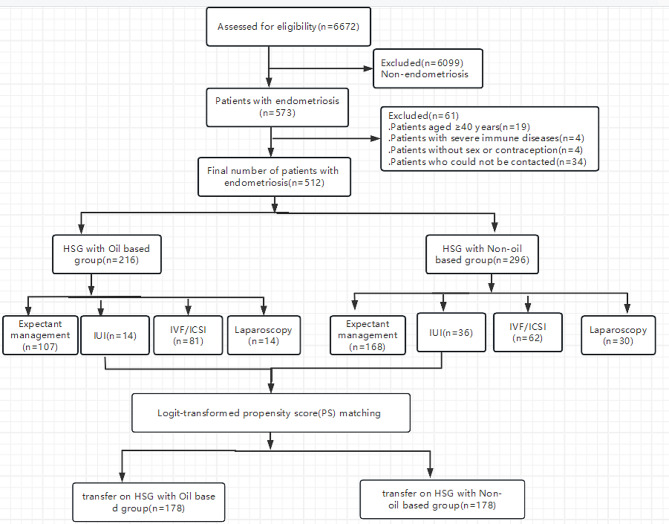
Flowchart of study recruitment and inclusion/exclusion criteria

This study enrolled a total of 512 patients, all of whom had infertility associated with endometriosis and underwent HSG between January 2020 and June 2022. Among them, 216 individuals received iodinated oil HSG, while 296 individuals received non-iodinated oil HSG. After logit-transformed PSM, 178 pairs were included into this study finally (Fig. 1).

Demographic characteristics before and after PSM were presented in Table 1. Of the cohort of 512 patients, PS matching was possible in 178 pairs (Table 1). PS matching reduced the standardized differences in baseline covariates between the Oil based group and the Non-oil based group.

**Table 1:** Patient characteristics before and after propensity score matching

	All patients (*n* = 512)			PS–matched Pairs (*n* = 356)		
	Oil based group	Non-oil based group	SMD	Oil based group	Non-oil based group	SMD
Parameter	*n* = 216	*n* = 296		*n* = 178	*n* = 178	
Age(year), Mean(SD)	32.00 (4.05)	31.86 (3.77)	0.036	31.63 (4.00)	31.44 (3.62)	0.052
Complication (%)			0.324			0.099
No complication	123 (56.9)	183 (61.8)		110 (61.8)	110 (61.8)	
Intrauterine adhesion	6 (2.8)	9 (3.0)		6 (3.4)	8 (4.5)	
Thyroid disease	14 (6.5)	19 (6.4)		11 (6.2)	8 (4.5)	
Chronic endometritis	12 (5.6)	8 (2.7)		7 (3.9)	7 (3.9)	
Adenomyosis	22 (10.2)	10 (3.4)		9 (5.1)	8 (4.5)	
Others	39 (18.1)	67 (22.6)		35 (19.7)	37 (20.8)	
BMI(kg/m^2^), Mean(SD)	21.37 (2.53)	20.97 (2.44)	0.16	21.08 (2.31)	20.99 (2.62)	0.036
Duration of infertility(year), Mean(SD)	2.96 (2.03)	2.84 (2.31)	0.055	2.89 (1.99)	2.43 (1.73)	0.243
Smoking, *n* (%)	4 (1.9)	5 (1.7)	0.012	2 (1.1)	3 (1.7)	0.048
Surgical history, *n* (%)			0.528			0.043
No	163 (75.5)	266 (89.9)		159 (89.3)	157 (88.2)	
Myoma/polyp resection/cystectomy	3 (1.4)	9 (3.0)		3 (1.7)	3 (1.7)	
Tubal surgery	43 (19.9)	14 (4.7)		12 (6.7)	14 (7.9)	
Cesarean section	7 (3.2)	4 (1.4)		4 (2.2)	4 (2.2)	
Others	0 (0.0)	3 (1.0)		0 (0.0)	0 (0.0)	
Total number of previous pregnancies resulting in live births, *n* (%)			0.168			0.064
0	182 (84.3)	249 (84.1)		150 (84.3)	154 (86.5)	
1	34 (15.7)	43 (14.5)		28 (15.7)	24 (13.5)	
2	0 (0.0)	4 (1.4)		0 (0.0)	0 (0.0)	
Miscarriage times, *n* (%)			0.198			0.124
0	171 (79.2)	237 (80.1)		145 (81.5)	150 (84.3)	
1	26 (12.0)	42 (14.2)		21 (11.8)	18 (10.1)	
2	14 (6.5)	16 (5.4)		11 (6.2)	10 (5.6)	
3	2 (0.9)	0 (0.0)		0 (0.0)	0 (0.0)	
4	2 (0.9)	1 (0.3)		1 (0.6)	0 (0.0)	
5	1 (0.5)	0 (0.0)		0 (0.0)	0 (0.0)	
Treatment after HSG, *n* (%)			0.401			0.569
Expectant management	107 (49.5)	168 (56.8)		102 (57.3)	124 (69.7)	
IUI	14 (6.5)	36 (12.2)		14 (7.9)	2 (1.1)	
IVF/ICSI	81 (37.5)	62 (20.9)		49 (27.5)	23 (12.9)	
Laparoscopy and/or Hysteroscopy	14 (6.5)	30 (10.1)		13 (7.3)	29 (16.3)	

### Odds ratio for the improvement of cumulative pregnancy rate in oil-based group vs. non-oil-based group

Comparing the Oil-based group to the Non-oil-based group, the fertility-enhancing impact of HSG was found to be more effective in the Oil-based group. Within 12 months after HSG, 111 out of 216 women in the Oil-based group (51.39%) had a clinical pregnancy, whereas 81 out of 296 women in the Non-oil-based group (27.36%) achieved a clinical pregnancy (OR, 2.81; 95% CI, 1.94 to 4.06). Additionally, the Oil-based group exhibited higher rates of live births at ≥ 28 weeks of gestation (68 (31.48%) vs. 59 (19.93%), OR: 1.85, 95% CI: 1.23 to 2.77) than the Non-oil-based group.

In the Oil-based group, the early miscarriage rate was 10 (4.6%), while in the Non-oil-based group, it was 6 (2.0%), with no statistically significant difference observed (OR, 0.43; 95% CI, 0.15 to 1.19). Similarly, there was no statistically significant difference between the two groups in terms of ectopic pregnancy rates (OR, 0.73; 95% CI, 0.15 to 3.64) (Table 2).

**Table 2:** Outcomes of the study 12 months after HSG

Outcome	Oil based group (*n* = 216)	Non-oil based group (*n* = 296)	OR (95%Cl)
Clinical pregnancy—no. (%)	111(51.39)	81(27.36)	2.81(1.94–4.06)
Live birth ≥ 28 wk of gestation—no./total no. (%)	68/216(31.48)	59/296(19.93)	1.85(1.23–2.77)
Early miscarriage—no. (%)	10(4.6)	6(2.0)	0.43(0.15–1.19)
Ectopic pregnancy—no. (%)	3(1.4)	3(1.0)	0.73(0.15–3.64)

### Associations between contrast medium and the clinical pregnancy in the crude analysis, multivariable analysis, and propensity-score analyses (Non-oil-based group vs. Oil based group)

Comparison with the Oil-based group, the Non-oil-based group exhibited an unadjusted model Odds Ratio (OR) of 0.38 (95%CI: 0.27–0.55, *p* < 0.001). In the multivariable analysis, the OR_95CI was 0.34 (0.22–0.51), *p* < 0.001. Employing the inverse probability of treatment weighting (IPTW) regression analysis, the OR_95CI was 0.39 (0.27–0.57), *p* < 0.001. Additionally, using propensity score matching, the OR_95CI was 0.22 (0.14–0.35), *p* < 0.001. All these statistical methods consistently demonstrated a higher clinical pregnancy rate in the Oil-based group (Table 3).

**Table 3:** Associations between contrast medium and the clinical pregnancy in the crude analysis, multivariable analysis, and propensity-score analyses (Non-oil-based group vs. Oil based group)

Analysis	OR_95CI	*P*_value
Crude analysis	0.38 (0.27 ~ 0.55)	< 0.001
Multivariable analysis^a^	0.34 (0.22 ~ 0.51)	< 0.001
Weighted.IPTW^b^	0.39 (0.27 ~ 0.57)	< 0.001
PropensityScore.Matched^c^	0.22 (0.14 ~ 0.35)	< 0.001

a. Shown is the odds ratio from the multivariable logistic model, with adjusted for all covariates in table (age, complication, BMI, duration of infertility, smoking status, surgical history, total number of previous, pregnancies resulting in live births, miscarriage times and treatment after HSG)

b. Shown is the primary analysis with multivariable logistic model with the same covariates with inverse probability weighting according propensity score

c. Shown is the odds ratio from the multivariable logistic model with the same strata and covariates with matching according to the propensity score. The analysis included 178 patients (178 undergoing HSG examination in the Oil based group and 178 in the Non-oil-based group)

### Cumulative clinical pregnancy rate

**Fig. 2:**
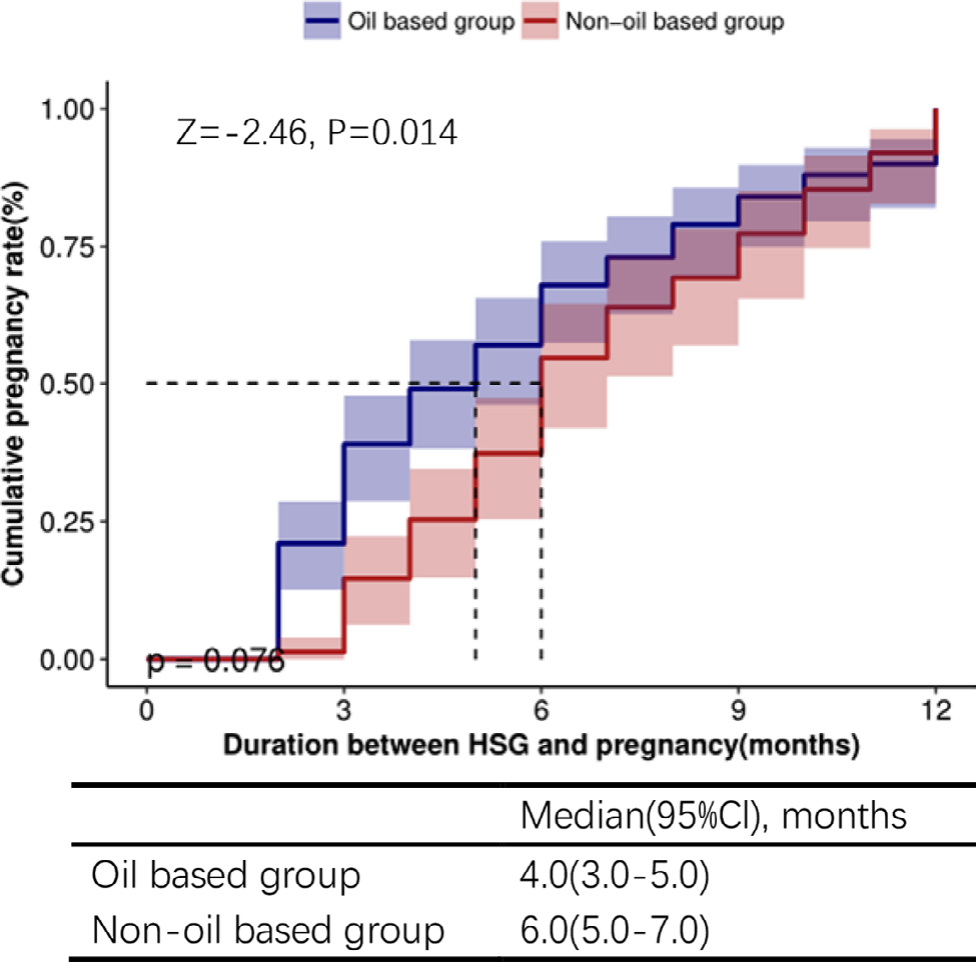
Cumulative pregnancy rate. Comparison of the cumulative pregnancy rate between the Oil based group and Non-oil-based group

The cumulative clinical pregnancy rate within 12 months after HSG was notably higher in the Oil-based group (51.39%) compared to the Non-oil-based group (27.36%). In the Oil-based group, the median time between HSG and pregnancy was 4.0 months (95% CI: 3.0–5.0 months), whereas in the Non-oil-based group, it was 6.0 months (95% CI: 5.0–7.0 months). The cumulative clinical pregnancy rate demonstrated a significant increase in the Oil-based group compared to the Non-oil-based group according to the rank sum test (*p* = 0.014, Fig. 2).

### Stratified analysis using additional factors

**Fig. 3:**
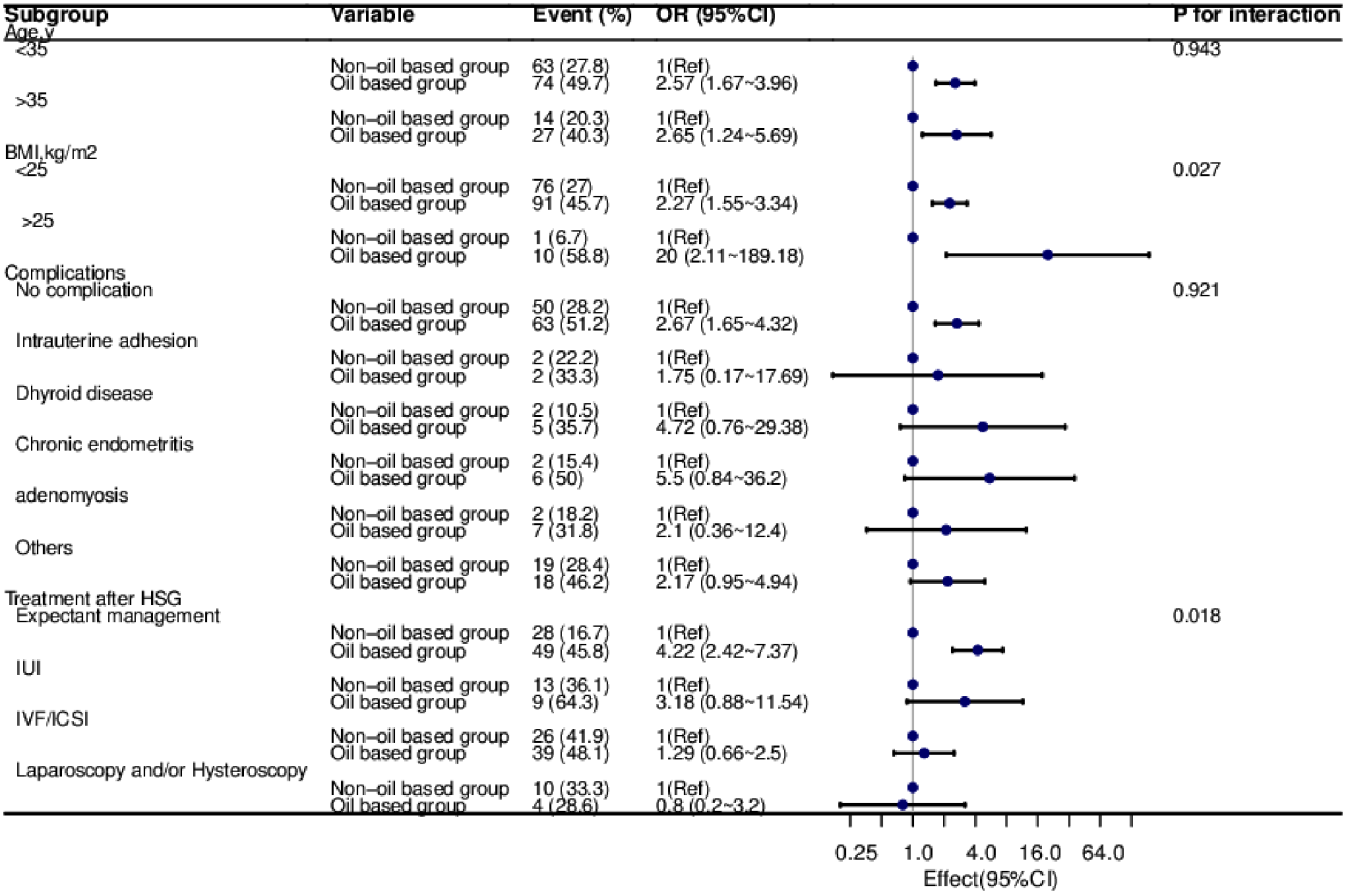
Relationship between cumulative pregnancy rate and the Oil based group and Non-oil-based group

To assess potential variations in the relationship between the contrast medium and pregnancy outcome, a stratified study was conducted in multiple subgroups. Stratification was performed based on age, BMI, complications, and treatment following HSG. Our findings indicate that the fertility-enhancing effect of the oil-based contrast agent is consistent across different age groups, BMI categories, the absence of complications, and among patients undergoing expectant treatment. Conversely, in patients with complications, those undergoing intrauterine insemination (IUI), in vitro fertilization (IVF), and combined laparoscopy/hysteroscopy procedures after the contrast agent administration, we did not observe the same fertility-enhancing effect,, as illustrated in Fig. 3.

## Discussion

In this retrospective investigation, we found that among infertile women affected by endometriosis who underwent HSG with oil contrast, the rate of clinical pregnancy within a year following the procedure was significantly higher compared to those who underwent the procedure without it. Additionally, women subjected to HSG with oil contrast exhibited a markedly elevated rate of subsequent live births.

Research has consistently demonstrated that the use of oil-based contrast agents during HSG contributes to improved fertility. Notably, findings from the water vs. oil (H2Oil trial) indicate that the oil-based group exhibited a higher rate of live births (38.8% vs. 28.1%) and sustained pregnancies (39.7% vs. 29.1%) compared to the water-based group [3]. Another Randomized Controlled Trial (RCT) reported that the oil group not only experienced a shorter time to pregnancy than the water group but also demonstrated a higher cumulative on-going pregnancy rate, on-going pregnancy within 6 months (29.1% vs. 20.1%), clinical pregnancy (39.5% vs. 29.1%), and live births at 24 weeks of gestation (36.1% vs. 27.7%), with all these differences being statistically significant (*P* < 0.01) [23].

The mechanisms through which iodinated oil enhances pregnancy rates remain elusive but are likely multifaceted. Proposed mechanisms can be categorized based on their sites of action, including the fallopian tubes, endometrium, and peritoneum. Firstly, fallopian tube flushing, which involves the mechanical removal of debris, mucus plugs, or clearance of adhesions around the fallopian tubes, may restore the patency of previously obstructed tubes [24, 25]. Secondly, oil-based contrast agents may enhance the receptivity of the endometrium. Some infertile women have successfully conceived after undergoing HSG with iodinated oil, even when the HSG results indicated tubal obstruction, suggesting that iodinated oil may not have penetrated the fallopian tubes or peritoneal cavity. This implies that iodinated oil may alter the uterine environment, improve endometrial receptivity, and subsequently increase embryo implantation rates.

Moreover, opiate alkaloids present in oil-based contrast agents derived from poppy seeds may interact with opiate receptors in the endometrium [26] or alter the uterine immunological response [7]. A randomized controlled animal study [27] demonstrated changes in the endometrial dendritic cell (DC) phenotype in mice following intrauterine injection of iodinated oil, with a decrease in CD205 + DCs and an increase in CD1 + DCs capable of presenting lipid antigens. This suggests a potential induction of non-specific antigen tolerance in the maternal uterine immune system. The third potential mechanism involves oil-based contrast agents forming a layer on macrophages, altering their shape and surface configuration, thereby reducing peritoneal macrophage phagocytosis and adhesion [28, 29].

Despite the established evidence demonstrating the superior fertility-enhancing impact of oil-based contrast agents over non-oil-based counterparts, there is a notable lack of discussion on how this enhancement specifically influences infertility in women affected by endometriosis. Our findings indicate that HSG employing oil-based contrast agents can significantly improve fertility in infertile patients with endometriosis. Several factors could elucidate these phenomena. Widely acknowledged, endometriosis is an inflammatory disease associated with immune dysregulation, where immune dysfunction is considered a pivotal factor in its development. Studies have consistently highlighted the presence of a robust immunosuppressive microenvironment in endometriosis [30], with local and systemic immune dysregulation contributing to the onset and progression of the condition [31–33].

Recent research further suggests a connection between endometriosis and alterations in both systemic and local immunity. Potential mechanisms include disturbances in the numbers and functions of neutrophils, monocytes/macrophages, dendritic cells (DCs), natural killer (NK) cells, and T cells [34]. Iodine, potentially one of the components in iodinated oil, emerges as a crucial factor responsible for these immunological changes in the endometrium, thereby potentially improving embryo implantation. The unique immunomodulatory properties of iodine may play a role in mitigating the immune dysregulation associated with endometriosis, contributing to the observed enhancement in fertility outcomes following HSG with oil-based contrast agents.

Research suggests a dichotomy in the impact of iodine on reproductive health, highlighting that high doses may be toxic, while low doses, as observed in rodent studies, could foster a uterine environment conducive to normal reproduction [35]. Iodinated oil, with its ability to enhance fertility in patients facing infertility combined with endometriosis, may operate by modifying the peritoneal and endometrial immune environments. Studies in mice have demonstrated that an excess of iodine can induce changes in the population of lymphocyte subsets [7, 36, 37]. Elevated iodine levels lead to an upregulation of Th17 cells, promoting inflammation, as evidenced in studies investigating the pathophysiology of autoimmune thyroiditis following an iodine load. Additionally, Treg cell suppression is identified as another contributing factor [36]. These findings suggest that iodine can alter the immune environment, potentially influencing embryo implantation.

Furthermore, a separate study [38] indicates that exogenous lipids injected into the peritoneal cavity are assimilated by dendritic cells (DCs), resulting in profound changes to the immunological environment of the peritoneal cavity. This immunological influence, driven by lipids, may pave the way for alternative treatment approaches for clinical disorders associated with immunological abnormalities in the peritoneal cavity, potentially fostering fertility. Lipiodol, derived from poppy seed oil, distinguishes itself from most other contrast media by its viscosity, non-ionic nature, and high iodine content (480 mg/ml). While various Water-Soluble Contrast Media (WSCM) used for HSG exhibit significant differences in ionicity, viscosity, hyperosmolarity, and iodine concentration, the role of iodine in enhancing fertility cannot be overlooked, particularly owing to the unusually high iodine concentration and prolonged half-life of Lipiodol.

This study’s innovation lies in presenting evidence for enhanced fertility in patients facing infertility coupled with endometriosis through iodinated oil HSG. Unlike previous Randomized Controlled Trials (RCT) that predominantly focused on the general infertility population, excluding specific groups like those with endometriosis or polycystic ovarian syndrome, this study demonstrates that, for individuals with infertility and endometriosis, oil-based contrast agents prove more effective in improving fertility compared to water-soluble contrast agents. Currently, endometriosis is estimated to contribute to 30-50% of infertility cases [12], with direct medical costs for women with endometriosis more than double that of women without this condition [39]. Addressing endometriosis-associated infertility is financially burdensome, and based on our study’s results, it is recommended to prioritize the use of oil-based contrast agents for patients with endometriosis and infertility undergoing HSG examinations to augment their pregnancy rates. Exploring potential regulatory effects of iodine oil on the endometrium and the intraperitoneal environment, future research for patients with infertility combined with endometriosis may contemplate investigating methods such as uterine cavity iodine infusion, paving the way for novel treatment modalities tailored to this specific patient group.

This study is not without limitations. Firstly, the sample size was relatively small, warranting validation of the findings in a larger survey with a more extensive participant pool. Secondly, patients with endometriosis are prone to developing comorbidities such as chronic endometritis, hyperthyroidism, and adenomyosis. Despite the exclusion of “female participants with uncontrolled endocrine disorders known to diminish natural pregnancy chances” in the inclusion criteria, the potential influence of these conditions on the study results cannot be entirely ruled out. Lastly, the retrospective nature of the study, despite stratified analyses, introduces the possibility of residual confounding effects due to unmeasured or unidentified factors. These limitations should be taken into consideration when interpreting the study’s findings.

## Conclusion

In summary, infertile patients with endometriosis undergoing HSG treatment with oil-based contrast agents exhibit a significant enhancement in fertility compared to those undergoing the procedure without oil-based contrast agents. The use of oil-based HSG may prove beneficial in treating infertility, especially in individuals with endometriosis, in addition to its diagnostic applications.

## Data Availability

No datasets were generated or analysed during the current study.

## Abbreviations

HSG: Hysterosalpingography
IUI: Intrauterine insemination
IVF: In vitro fertilization
ICSI: Intracytoplasmic sperm injection
CI: Confidence interval
OR: Odds ratio
IVF: In vitro fertilization
RIF: Recurrent implantation failure
RCT: Randomized controlled trial
PSM: Propensity score matching
